# Genomic and Epidemiological Features of Two Dominant Methicillin-Susceptible Staphylococcus aureus Clones from a Neonatal Intensive Care Unit Surveillance Effort

**DOI:** 10.1128/msphere.00409-22

**Published:** 2022-10-11

**Authors:** Medini K. Annavajhala, Nicole E. Kelly, Wenjing Geng, Samantha A. Ferguson, Marla J. Giddins, Emily C. Grohs, Alexandra Hill-Ricciuti, Daniel A. Green, Lisa Saiman, Anne-Catrin Uhlemann

**Affiliations:** a Department of Medicine, Division of Infectious Diseases, Columbia University Irving Medical Center, New York, New York, USA; b Department of Pediatrics, Columbia University Irving Medical Center, New York, New York, USA; c Neonatal Center, Beijing Children’s Hospital, Capital Medical University, National Center for Children’s Health, Beijing, China; d Clinical Microbiology Laboratory, Department of Pathology & Cell Biology, Columbia University Irving Medical Center, New York, New York, USA; e Department of Infection Prevention and Control, New York-Presbyterian Hospital, New York, New York, USA; University of Nebraska Medical Center

**Keywords:** methicillin-susceptible *Staphylococcus aureus*, neonatal intensive care unit (NICU), mupirocin resistance, genomic surveillance, MSSA, NICU

## Abstract

Methicillin-susceptible Staphylococcus aureus (MSSA) is a more prevalent neonatal intensive care unit (NICU) pathogen than methicillin-resistant S. aureus (MRSA). However, the introduction and spread of MSSA, the role of systematic decolonization, and optimal infection prevention and control strategies remain incompletely understood. We previously screened infants hospitalized in a university-affiliated level III to IV NICU twice monthly over 18 months for S. aureus colonization and identified several prevalent staphylococcal protein A (*spa*) types. Here, we performed whole-genome sequencing (WGS) and phylogenetic comparisons of 140 isolates from predominant *spa* types t279, t1451, and t571 to examine possible transmission routes and identify genomic and epidemiologic features associated with the spread of dominant clones. We identified two major MSSA clones: sequence type 398 (ST398), common in the local community, and ST1898, not previously encountered in the region. ST398 NICU isolates formed distinct clusters with closely related community isolates from previously published data sets, suggesting multiple sources of acquisition, such as family members or staff, including residents of the local community. In contrast, ST1898 isolates were nearly identical, pointing to clonal expansion within the NICU. Almost all ST1898 isolates harbored plasmids encoding mupirocin resistance (*mupA*), suggesting an association between the proliferation of this clone and decolonization efforts with mupirocin. Comparative genomics indicated genotype-specific pathways of introduction and spread of MSSA via community-associated (ST398) or health care-associated (ST1898) sources and the potential role of mupirocin resistance in dissemination of ST1898. Future surveillance efforts could benefit from routine genotyping to inform clone-specific infection prevention strategies.

**IMPORTANCE** Methicillin-susceptible Staphylococcus aureus (MSSA) is a significant pathogen in neonates. However, surveillance efforts in neonatal intensive care units (NICUs) have focused primarily on methicillin-resistant S. aureus (MRSA), limiting our understanding of colonizing and infectious MSSA clones which are prevalent in the NICU. Here, we identify two dominant colonizing MSSA clones during an 18-month surveillance effort in a level III to IV NICU, ST398 and ST1898. Using genomic surveillance and phylogenetic analysis, coupled with epidemiological investigation, we found that these two sequence types had distinct modes of spread, namely the suggested exchange with community reservoirs for ST398 and the contribution of antibiotic resistance to dissemination of ST1898 in the health care setting. This study highlights the additional benefits of whole-genome surveillance for colonizing pathogens, beyond routine species identification and genotyping, to inform targeted infection prevention strategies.

## INTRODUCTION

Staphylococcus aureus is an important cause of morbidity and mortality in both adult and pediatric populations (1–4), including infants hospitalized in neonatal intensive care units (NICUs) (5, 6). In neonates, the acquisition and spread of S. aureus can occur through multiple routes, including during delivery and from close contact with parents and health care providers (5, 7–10). While methicillin-susceptible S. aureus (MSSA) is responsible for a larger proportion of invasive infections than methicillin-resistant S. aureus (MRSA) (6, 11), NICU surveillance and infection control efforts have largely prioritized MRSA colonization, the major risk factor for infection (12, 13). Active surveillance for MSSA colonization in the NICU setting is only infrequently being implemented and has shown different patterns of clonal dominance versus diversity (10, 14–17). Thus, colonization rates, routes of introduction, and potential transmission of MSSA in the NICU are incompletely understood, and optimal MSSA infection prevention and control strategies remain unclear.

We recently undertook a surveillance effort to elucidate the prevalence and diversity of S. aureus colonization in a level III to IV NICU located in northern Manhattan, NY (18). As previously reported, colonization by MSSA (13.6%) was markedly higher than colonization by MRSA (2.4%) (18). Of the diverse MSSA staphylococcal protein A (*spa*)-types identified during the surveillance period, t279, t1451, and t571 were highly prevalent, accounting for almost one-third of NICU MSSA isolates (18). MSSA sequence type 398 (ST398), encompassing *spa*-t1451 and t571, has been identified in populations within Europe, China, the Caribbean, and New York, specifically, northern Manhattan (19–25). ST398 also accounts for 13 to 14% of NICU MSSA colonization based on surveillance efforts in Clamart, France, and Beijing, China (26, 27). In contrast, MSSA ST1898, which includes *spa*-t279, has not been extensively described and is rarely reported in the NICU (28). Therefore, this surveillance effort represents a unique opportunity to study the epidemiology and genomic characteristics of this sequence type.

In this study, we apply whole-genome sequencing (WGS) to identify prominent colonizing NICU MSSA clones identified in the prior surveillance effort at our hospital center, as well as factors associated with their success. Using phylogenetic analysis and epidemiological approaches, we show that the most prevalent MSSA sequence types, ST398 and ST1898, were characterized by distinct routes of acquisition and spread. The use of long-read sequencing was also leveraged to elucidate the potential role of antibiotic resistance in the spread of the mupirocin-resistant ST1898 in particular. These results highlight the ability of genomic surveillance to elucidate the evolutionary and epidemiological trajectories of MSSA in the NICU, ultimately informing targeted mitigation strategies to reduce its spread.

## RESULTS

### Genomics of prevalent NICU MSSA *spa* types.

Over the 18-month NICU surveillance effort, 125 MSSA isolates from 66 patients underwent WGS, representing three highly prevalent *spa* types (t279, t1451, and t571). MSSA with t1451 and t571 belonged to sequence type ST398 (*n* = 31 from 18 infants), a common MSSA previously identified in the northern Manhattan community, through household sampling (19, 25). Isolates with the dominant *spa* type, t279, were identified as belonging to ST1898 (*n* = 94 from 48 infants), which was not encountered in diverse community sampling (19, 24, 25, 29–31). In order to determine epidemiological and molecular factors enabling the persistent prevalence of these two MSSA clones in the NICU, we investigated the phylogenetic relationships and genomic characteristics of both ST398 and ST1898.

### Epidemiology of MSSA ST398 in the NICU.

**FIG 1:**
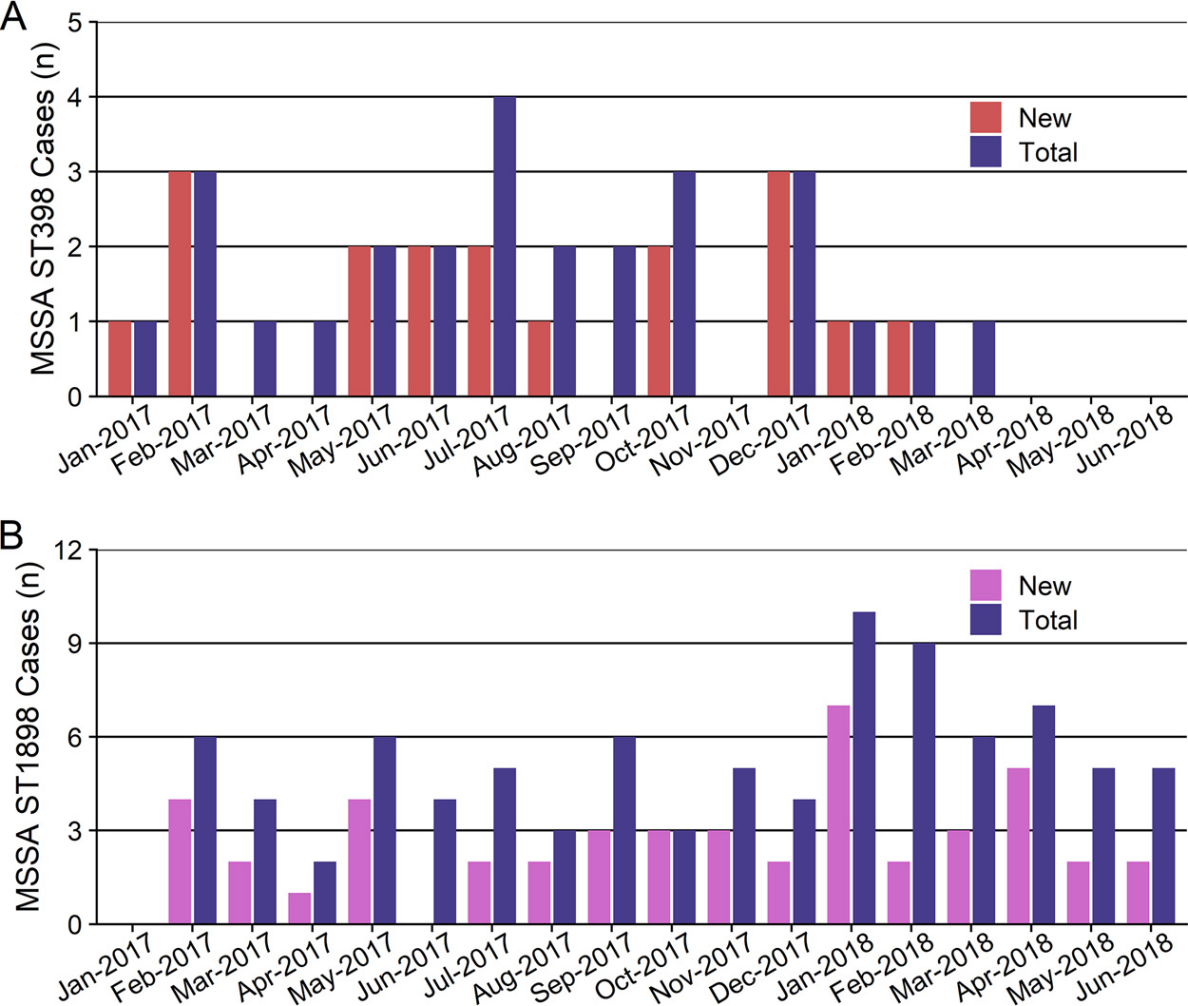
ST398 and ST1898 epidemiologic curves during an 18-month NICU surveillance effort. (A) Number of newly identified infants with ST398 (harboring *spa* types 1451 or 571) (red) and total number of ST398 cases per month (blue). There were multiple months with no new cases of ST398, and no ST398 isolates were identified beyond April 2018. (B) Number of newly identified infants with ST1898 (purple) and total number of ST1898 (harboring *spa* t279) cases per month (blue). In every month of the surveillance period, except January and June 2017, one to eight new cases of ST1898 were identified.

Among 271 MSSA-positive infants identified during the surveillance effort, 18 (7%) were colonized by ST398, 12/18 (67%) with *spa* type t1451 and 6/18 (33%) with t571 (Fig. S1). Of these 18 infants, 5 (28%) also had one or more subsequent positive cultures for other S. aureus sequence types. The median time between admission and first positive MSSA ST398 culture was 32 days (interquartile range [IQR], 16.5 to 40.5 days). The ST398 epidemiologic curve revealed a sporadic distribution of acquisition with no clear clustering (Fig. 1A). The overall colonization rate of ST398 was 1.2 per 100 NICU admissions, with one to three incident cases occurring during each of 10 months within the 18-month study period.

### Community-associated clone ST398 in the NICU and surrounding community.

To investigate potential routes of acquisition for MSSA ST398, including from outside the NICU, we performed phylogenetic reconstruction of the 31 ST398 isolates from our surveillance effort, encompassing *spa* t1451 and t571, along with 271 MSSA ST398 isolates, all *spa* t571, from prior household sampling in the surrounding northern Manhattan community (25). Pairwise distances between isolates ranged from 0 to 298 single nucleotide polymorphisms (SNPs) (median, 78 SNPs).

**FIG 2:**
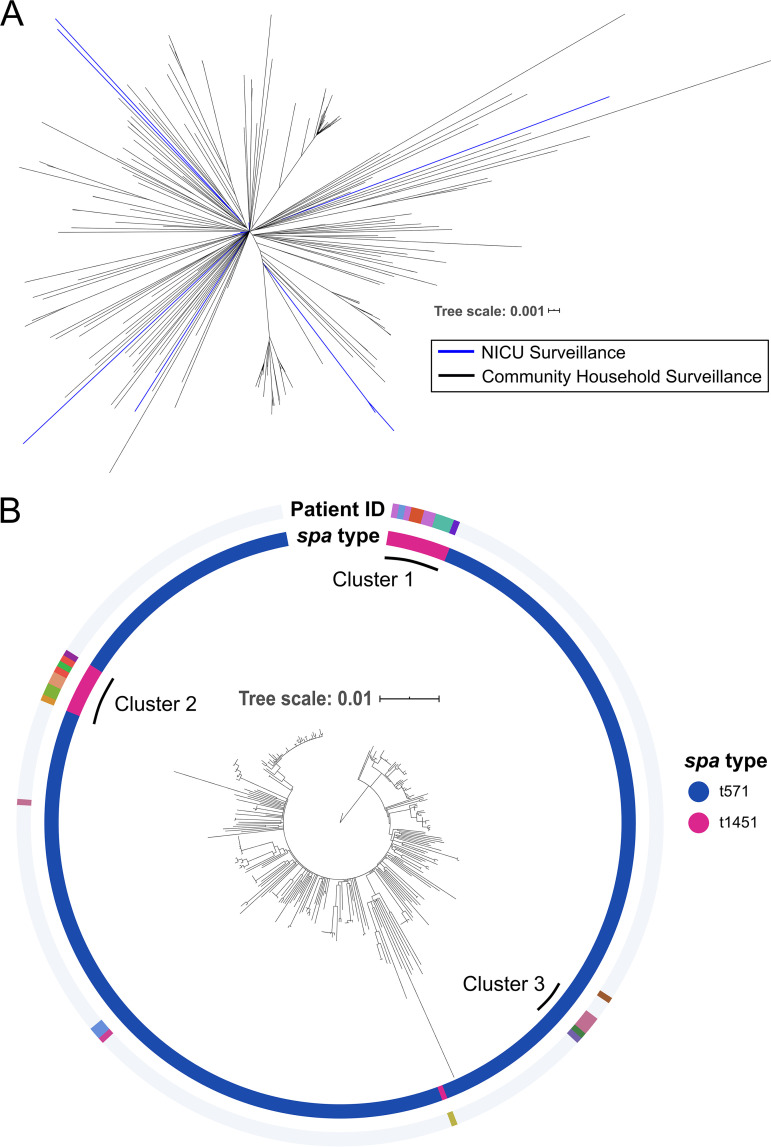
Phylogenetic tree of NICU and local community MSSA ST398. MSSA isolates from our NICU which were identified as ST398 using WGS (*n* = 31) were mapped against a previously published ST398 reference genome (GenBank CP003045) isolated from within the local community, along with publicly available short reads from 271 ST398 isolates also collected from our local household study (25). Concatenated SNPs covering the core chromosome of the reference genome totaled 5,338 bp, with pairwise distances between isolates ranging from 0 to 298 SNPs (median, 78 SNPs). Maximum-likelihood phylogenetic reconstruction was performed using RAxML with 100 bootstrap replicates (48). (A) Unrooted phylogenetic tree of all NICU (blue branches) and community (black branches) MSSA ST398 isolates reveals that many NICU isolates cluster with community isolates, suggesting possible introduction of these isolates from the community rather than the NICU. (B) The MSSA ST398 phylogeny, rooted at the reference isolate. The inner ring denotes isolate *spa* type, and colors along the outer ring represent individual infants to whom each NICU isolate belongs. The largest NICU cluster, comprising 11 isolates from 5 patients, is marked cluster 1 and was used for targeted transmission analysis (Fig. 3).

Phylogenetic reconstruction of ST398 isolates revealed close relationships between NICU isolates from the current study period and community MSSA ST398 collected between April 2005 and March 2013 (Fig. 2A) (25). NICU isolates from 2 infants were within 20 core genome SNPs of at least 1 community isolate, despite a gap of 4 to 12 years between these collections, suggesting multiple instances of acquisition via community reservoirs of ST398. The 31 NICU MSSA ST398 isolates differed by a median of 101 pairwise SNPs (IQR, 51 to 113 SNPs) (see Fig. S2A in the supplemental material). However, the majority (*n* = 24/31, 77%) formed 3 distinct clusters (clusters 1, 2, and 3)—defined as isolates from 2 or more infants within a 15-SNP pairwise distance—with bootstrap support of 92%, 87%, and 79%, respectively (Fig. 2B). The remaining isolates were interspersed with previously collected community MSSA ST398 isolates across the phylogenetic tree. Cluster 1 consisted of 11 ST398-t1451 isolates from 5 different infants, cluster 2 had 9 ST398-t1451 isolates from 6 infants, and cluster 3 contained 4 ST398-t571 isolates from 2 infants. Isolates from different infants were significantly more closely related within versus across clusters, providing further evidence for the three distinctly clustered groups (Fig. S2B). However, each NICU cluster was still relatively closely related to community MSSA in terms of median pairwise SNP distances between NICU and community isolates (64 SNPs for clusters 1 and 2, 14 SNPs for cluster 3).

10.1128/msphere.00409-22.3 FIG S2 Pairwise SNP distances between ST398 or CC15-t279 isolates. (A) Density plot of pairwise SNP distances between all pairs of ST398 isolates sequenced with WGS (*n* = 31) compared to the reference genome CP003045. Pairwise distances were binned by intervals of 10 SNPs for visualization. (B) Violin plot displaying pairwise SNP distances between ST398 isolates collected from different infants, stratified by whether isolate pairs were either located in the same phylogenetic cluster (“within cluster X”) or in different or no phylogenetic clusters (“across clusters”) (see Fig. 2B). (C) Density plot of pairwise SNP distances between all pairs of CC15-t279 isolates sequenced with WGS (*n* = 94) and aligned against the index CUMC4945 isolate collected in 2016 (NCBI genome accession number JAEHFK000000000). (D) Violin plot displaying pairwise SNP distances between CC15-t279 isolates collected from the same (“within patient”) or different patients (“across patient”). Download FIG S2, TIF file, 0.6 MB. Copyright © 2022 Annavajhala et al. 2022 Annavajhala et al. https://creativecommons.org/licenses/by/4.0/ This content is distributed under the terms of the Creative Commons Attribution 4.0 International license.

**FIG 3:**
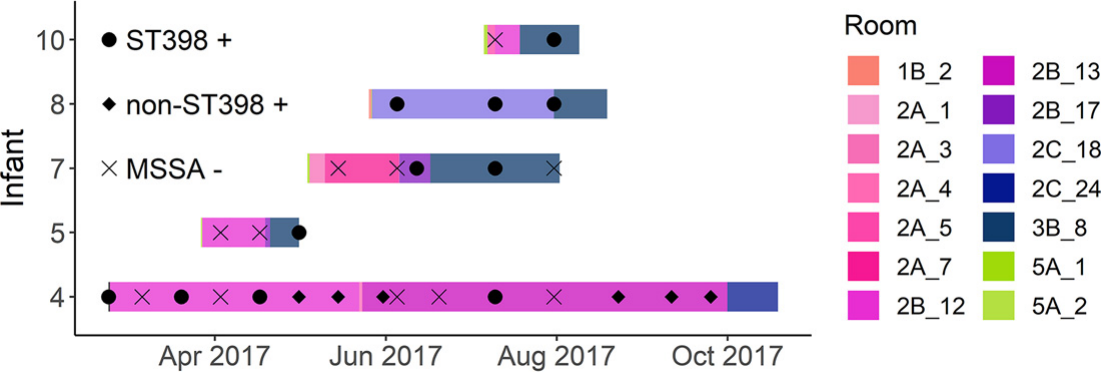
Contact tracing for MSSA ST398 *spa* type 1451 phylogenetic cluster. Temporal and spatial distribution of a subset of infants harboring MSSA ST398, identified as cluster 1 (Fig. 2). Whole-genome sequencing (WGS) revealed that 11 ST398 isolates from the 5 infants shown, all *spa* type 1451, were phylogenetically more closely related than other isolates (median pairwise distance, 6 SNPs). Rooms within the NICU are coded by color, with similar shades corresponding to different units. ST398 cultures (circles), non-ST398 MSSA cultures (diamonds), and negative surveillance swabs (Xs) are shown for each infant throughout the study period. Figure S1 shows the spatiotemporal distributions for all infants who harbored MSSA ST398 throughout the 18-month surveillance.

The close pairwise SNP distances within each cluster of isolates from different infants indicated that some patient-to-patient transmission within the NICU was likely. We therefore used genomic data to assess possible NICU locations associated with acquisition of ST398, by performing contact tracing within phylogenetic clusters (Fig. S3). We focused on the 5 infants linked to the 11 isolate ST398-t1451 cluster 1 isolates (Fig. 3), since this cluster was distinctly separated from other NICU and community ST398 isolates (Fig. 2B).

10.1128/msphere.00409-22.4 FIG S3 MSSA ST398 heat map. Temporal and spatial distribution of all infants who harbored MSSA ST398 during our 18-month surveillance effort. Each row represents a unique infant. Rooms within each NICU unit are coded by color, with similar shades corresponding to the same units (e.g., unit 2A, containing rooms 1 to 10). Positive ST398 cultures are represented by circles displayed at the date of culture collection. Download FIG S3, TIF file, 0.2 MB. Copyright © 2022 Annavajhala et al. 2022 Annavajhala et al. https://creativecommons.org/licenses/by/4.0/ This content is distributed under the terms of the Creative Commons Attribution 4.0 International license.

10.1128/msphere.00409-22.2 FIG S1 Study flowchart. A previously published study by Grohs et al. (18) assessed the use of surveillance swabs to identify MSSA colonization and diversity in neonates over an 18-month period, January 2017 to June 2018. During the surveillance period, 1,556 infants were admitted to the NICU. Of these, 271 total infants were found to harbor MSSA based on either surveillance swabs or clinical isolates collected as part of routine care. In total, 94 isolates from 48 infants with *spa* t279 and 31 isolates from 18 infants with *spa* t1451 or t571 collected during the surveillance timeframe underwent WGS for this study. In addition, we identified and whole-genome sequenced 15 clinical isolates from 13 patients collected as part of routine care, belonging to t279 and closely related t228 from before the surveillance study. Download FIG S1, TIF file, 0.2 MB. Copyright © 2022 Annavajhala et al. 2022 Annavajhala et al. https://creativecommons.org/licenses/by/4.0/ This content is distributed under the terms of the Creative Commons Attribution 4.0 International license.

Infant 4, who was transferred from an outside institution and tested positive for ST398-t1451 on admission, had the earliest cluster 1 isolate. Despite multiple decolonization attempts, this infant continued to screen positive for ST398-t1451 over their year-long admission. After sharing a room with infant 4, infant 5 also screened positive for cluster 1-type ST398-t1451. Similarly, after sharing a room with infant 8, who harbored cluster 1 ST398-t1451, infant 10 became colonized. Infant 7 had temporal, but not spatial, overlap with other infants in this cluster and had ST398-t1451 identified by their first surveillance culture.

### NICU MSSA burden of S. aureus ST1898.

ST1898 was cultured from 48 (18%) of 271 MSSA-positive infants during the surveillance period, all of whom harbored *spa* type t279, with the exception of 1 infant with a nontypeable isolate. The median time between admission and first positive ST1898 culture was 44 days (IQR, 25 to 66 days). ST1898 acquisition persisted throughout the 18 study months after initial identification of this clone in February 2017 (Fig. 1B). The overall colonization rate of ST1898 was 3.1 per 100 NICU admissions (median of 2 and maximum of 7 incident cases per month). Infants with ST1898 were primarily housed in units 2A, 2B, and 2C, located on the same floor within the NICU (Fig. S4).

10.1128/msphere.00409-22.5 FIG S4 MSSA ST1898 heat map. Temporal and spatial distribution of all infants who harbored MSSA ST1898 in our NICU. Each row represents a unique infant. Rooms within each NICU unit are coded by color, with similar shades corresponding to the same units (e.g., unit 2A, containing rooms 0 to 10). Positive ST1898 cultures are represented by circles displayed at the date of culture collection. Download FIG S4, TIF file, 0.5 MB. Copyright © 2022 Annavajhala et al. 2022 Annavajhala et al. https://creativecommons.org/licenses/by/4.0/ This content is distributed under the terms of the Creative Commons Attribution 4.0 International license.

**FIG 4:**
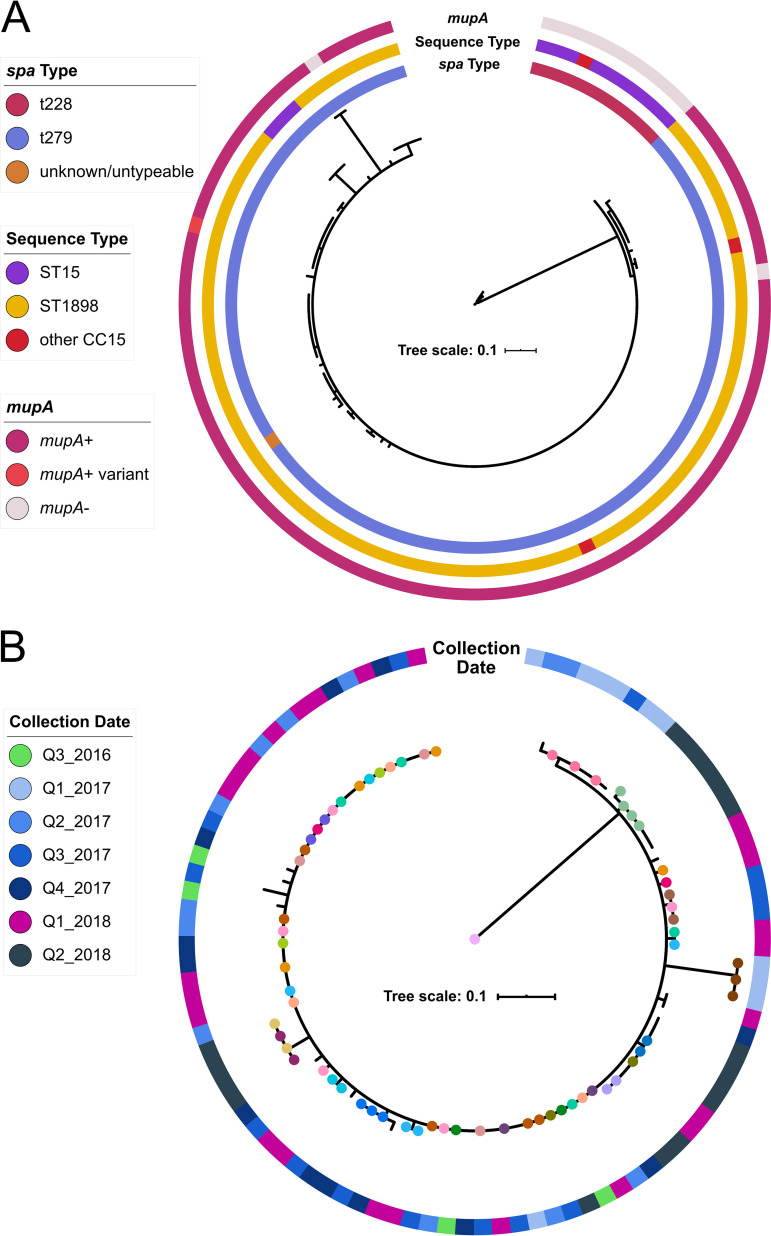
Phylogenetic reconstruction of NICU clonal complex CC15. WGS was performed on 106 CC15 isolates collected from our NICU, belonging to sequence types ST1898 and ST15. (A) The 106 CC15 isolates clustered by both *spa* type (inner ring) and sequence type (ST, middle ring). Screening for the high-level mupirocin resistance gene *mupA* (also referred to as *ileS*-2) revealed that this gene was only present in CC15-t279 isolates, with one CC15-t279 isolate containing a *mupA* variant allele and two CC15-t279 isolates missing the *mupA* gene. (B) We next generated a phylogenetic tree specifically for the 94 CC15-t279 isolates. Serial isolates from the same infant (colored dots on branch ends; no dot indicates singleton isolates) did not necessarily cluster together, and some isolates from vastly separated collection dates (outer ring) were closely related, highlighting the close relationships between CC15-t279/ST1898 isolates and indicative of local proliferation within the NICU.

To determine the full evolutionary history of ST1898 in the NICU, we sequenced 94 isolates collected during the 18-month surveillance period (t279 *n* = 93, unknown *spa* type *n* = 1) and 15 clinical isolates harboring either t279 or t228, which collectively belong to clonal complex CC15, collected in the year prior to the NICU MSSA surveillance effort (t279 *n* = 4, t228 *n* = 11). The index (earliest identified) NICU ST1898 isolate, CUMC4945, collected in August 2016, was used as a genomic reference. CC15 isolates were more closely related than ST398 isolates, with pairwise distances across all CC15 isolates ranging from 0 to 48 SNPs (median, 2 SNPs). Distinct clustering by *spa* type t228 versus t279 was also evident, (Fig. 4A), with a median of 32 SNPs (range, 29 to 48 SNPs) separating CC15-t228 and CC15-t279. Sequence types were associated stably with *spa* type, with 91/94 (97%) t279 isolates belonging to ST1898, a single-locus variant (SLV) of the t228-associated ST15; both ST15 and ST1898 belong to the clonal complex CC15.

Almost all CC15-t279 isolates (*n* = 89/94, 95%) were closely related, within 5 SNPs of each other (Fig. 4B). Most CC15-t279 isolate pairs were within one pairwise SNP, including those from different infants, and the maximum pairwise SNP distance between isolates from the same infant was two SNPs (Fig. S2C and D). A subset of 8 isolates from January to March 2017 (Q1_2017) and of 13 isolates collected from April to June 2018 (Q2_2018) were colocated on the phylogenetic tree. Additionally, there were many instances of isolates from different time periods clustering closely together; 88% (45/51) of infants were colonized with CC15-t279 isolates within ≤2 core genome SNPs of the index isolate, CUMC4945, despite having been collected a median of 490 days (IQR, 323 to 584 days) after CUMC4945.

### Mupirocin resistance in CC15.

During the surveillance period, eligible infants with positive MSSA or MRSA surveillance cultures were decolonized with mupirocin applied to the anterior nares and/or chlorhexidine baths (18). Infants with mupirocin-resistant isolates did not receive mupirocin. Overall, mupirocin resistance (MIC, ≥1,024 μg/mL) was noted in 10 MSSA *spa* types, including t279. All ST398 surveillance isolates were susceptible to mupirocin, and had resistance to erythromycin and clindamycin as observed in MSSA-ST398 from the northern Manhattan community surrounding the NICU (19, 25). The majority of t279 isolates (96%, 88/92 tested) were mupirocin resistant.

We used long-read sequencing of the index ST1898 CUMC4945 to identify genomic features which could be associated with the spread of this clone, including mupirocin resistance. The index isolate genome included three genomic islands (15,351, 7,309, and 5,026 bp) encoding, respectively, a type VII secretion system, proteins involved in stress response and cellular growth, and camphor resistance (*crcB*). CUMC4945 also harbored one integrated prophage, the β-hemolysin-converting staphylococcal phage φNM3 common to human S. aureus isolates (32). No ST1898 isolates, nor any NICU or community ST398 isolates, harbored *qacAB* genes, which are associated with chlorhexidine tolerance (33, 34).

The majority of ST1898 isolates also contained a 32,203-bp pCUMC4945_1 plasmid (complete circularized contig CUMC4945_13 in assembly JAEHFK000000000). This plasmid consists of two distinct segments which each aligned to other S. aureus plasmid sequences available in NCBI databases (Fig. S5). This suggests pCUMC4945_1 as a novel hybrid plasmid comprising segments derived from previously identified S. aureus plasmids. Segment A, the 21.7-kbp backbone of pCUMC4945_1, includes the S. aureus β-lactamase-encoding *Tn*552 transposon, bacteriocin and lactococcin resistance proteins, and the cadmium resistance genes *cadDX*. Segment B (10.5 kbp) encodes an operon including a multidrug resistance transporter protein along with the *mupA* gene, associated with high-level mupirocin resistance. The presence of *mupA* was stably associated with ST1898 NICU isolates (present in 92/94 t279 isolates, 98%) and not found in t228 isolates (Fig. 4A). Neither NICU nor community household MSSA ST398 isolates contained *mupA*, consistent with their susceptibility to mupirocin (18, 25). CC15-t228 isolates collected prior to the NICU surveillance effort harbored plasmids containing the segment A backbone of pCUMC4945_1, but not the *mupA*-encoding segment B (Fig. S5).

10.1128/msphere.00409-22.6 FIG S5 Alignment of NICU CC15 isolates against pCUMC4945_1. The resolved pCUMC4945_1 contig from the CUMC4945 hybrid assembly reference genome (contig number 13) was used as the reference for alignment of short reads from all 106 NICU CC15 isolates. The 32,203-bp pCUMC4945_1 sequence is shown at the top, with several key gene clusters annotated. The blue dendrogram on the left shows the maximum-likelihood tree structure (no branch lengths) after 100 bootstrap replicates. Gray rectangles indicate 100% sequence homology; black vertical lines or rectangles indicate individual SNPs or SNP clusters; horizontal black lines indicate no alignment. Alignment of pCUMC4945_1 using BLASTn revealed that the full plasmid sequence had not been previously described in the nucleotide (nt) database; rather, two distinct segments were apparent: segment A (~21-kbp segment), which aligned to unnamed plasmids found in several S. aureus clinical strains (e.g., UP_620, GenBank accession number CP047844; BPH2760, LR130510), and segment B (~11 kbp), which includes the mupirocin resistance-conferring pCUMC4945_1-*mupA* gene on a putative transposable element similar to publicly available plasmid sequences from not only S. aureus (SAP073A, GQ900425) but also Staphylococcus haemolyticus (FDAARGOS_517, CP033813), Staphylococcus epidermidis (SAP045A, GQ900402), and Staphylococcus lugdunensis (Tlug63N-4 plasmid pT63N, KU882686). Download FIG S5, TIF file, 1.9 MB. Copyright © 2022 Annavajhala et al. 2022 Annavajhala et al. https://creativecommons.org/licenses/by/4.0/ This content is distributed under the terms of the Creative Commons Attribution 4.0 International license.

## DISCUSSION

Surveillance efforts, including our prior work, have shown that diverse MSSA *spa* types are found in the NICU, yet dominant MSSA clones can arise (17, 18). Taking advantage of bacterial genomics, here, we demonstrate the distinct, clone-specific epidemiology of MSSA genotypes prevalent in the NICU. Our data suggest two distinct transmission modes: circulation of isolates between the community and NICU, indicative of multiple acquisition events and likely community reservoirs, versus a health care-adapted clone, characterized by widespread proliferation within the NICU and high-level mupirocin resistance. These two different modes of introduction and spread could have significant practical implications for infection prevention and control practices in the NICU.

During an 18-month surveillance period, ST398 and ST1898 were dominant clones of MSSA in the NICU (18). Notably, the presence of two predominant clones in our hospital center NICU contrasts with a recent NICU MSSA surveillance effort in Germany, which observed diverse *spa* types at relatively similar frequencies (17). We therefore hypothesize that genomic factors and transmission patterns unique to ST398 and ST1898, as outlined in this study, may have contributed to their success relative to other *spa* types identified at our center.

In both adult and pediatric settings, ST398 has been identified as a highly transmissible and prevalent colonizing MSSA clone (26, 27). Indeed, we previously found that ST398 made up a significant proportion of MSSA cultures in the northern Manhattan community surrounding our NICU (19, 20). Multiple acquisition events of ST398 occurred throughout the study period in our NICU population. Contact tracing informed by genomics revealed evidence of transmission among infants who overlapped spatially but not temporally, suggesting environmental reservoirs that potentially contributed to ST398 acquisition, as had been shown for households in the local community (25). Furthermore, infants were hospitalized in the NICU for an average of over 1 month before screening positive for ST398 colonization, suggesting numerous opportunities for acquisition from the NICU environment or repeated contact with visitors. These observations raise the possibility of persistence of S. aureus on patient care equipment and high-touch surfaces and/or transmission from family members (35, 36).

MSSA ST1898, the dominant clone in this surveillance effort, has not been well-described in the literature and was rarely reported in prior NICU surveillance efforts (28). The persistence of ST1898 throughout the 18-month surveillance period, the close pairwise distances between isolates, and the phylogenetic interspersing of isolates from different infants, coupled with strong epidemiological links between infants carrying CC15-t279 isolates (18), suggest local NICU proliferation as the likely source for this dominant clone. These data suggest localized spread within the NICU over a sustained time period, likely linked to either infant-to-infant transmission or multiple, frequent acquisition events from unidentified common reservoirs such as shared personnel, equipment, or surfaces. Moreover, ST1898 has not been previously identified during community sampling in neighborhoods surrounding our NICU center, making introductions into the NICU from community members unlikely.

Both ST1898 and ST398 persisted in the NICU despite MSSA decolonization protocols implemented as an important component of our surveillance program to reduce the risk of invasive infections. We identified multiple instances in which infants had two consecutive positive cultures for ST398 despite decolonization, suggesting that either (i) infants can effectively be decolonized but quickly become recolonized or (ii) decolonization efforts are not effective at reaching all colonized sites. For example, mupirocin and external chlorhexidine gluconate (CHG) baths do not reach the oropharynx, a significant site of S. aureus colonization (37). Moreover, the previously undescribed plasmid, pCUMC4945_1, containing mupirocin and other resistance genes, points to the role of antimicrobial pressure in enabling the proliferation of ST1898. The presence of the pCUMC4945_1 segment A backbone without *mupA*-containing segment B in early CC15-t228 isolates also suggests that this unique plasmid may have arisen in CC15-t279 and ST1898 as a result of selective pressure from decolonization efforts. To date, we have not found evidence of its spread to other MSSA clones.

Importantly, this study had several limitations to consider. As this was a retrospective study conducted after the conclusion of an 18-month NICU S. aureus surveillance effort, these results did not inform patient care or infection prevention and control measures. Future surveillance strategies assessing the use of real-time genomic typing to inform epidemiological interventions will be key in understanding how sequencing data can be integrated with infection control efforts in the NICU. Furthermore, our spatiotemporal contact tracing, even after accounting for phylogenetic clustering of isolates, did not address all potential aspects of S. aureus transmission. Culturing infants’ mothers and other visitors, NICU room surfaces and patient care equipment, and staff who have regular contact with infants may allow for a better understanding of the modes of acquisition.

### Conclusions.

In the current study, we leveraged short- and long-read WGS and phylogenetics to identify potential factors enabling the success of dominant NICU MSSA clones ST398 and CC15. We discerned the distinct genomic epidemiology of sequence type ST398, which had strong phylogenetic associations with MSSA isolates from the local community, contrasted with clonal complex CC15, which exhibited localized proliferation within the NICU despite not being significantly described in other prior NICU surveillance efforts. Our results indicate that institution of routine MSSA surveillance coupled with WGS genotyping may be useful to both rapidly identify or rule out MSSA outbreaks and differentiate probable sources of acquisition for individual MSSA clones. Genomic surveillance could thereby inform appropriately tailored interventions to mitigate MSSA infection and spread in NICU settings.

## MATERIALS AND METHODS

### NICU surveillance for colonizing S. aureus.

All infants hospitalized in a 58-bed university-affiliated level III to IV NICU with ~1,000 admissions per year located in northern Manhattan were screened twice monthly over an 18-month period, and/or upon admission, as previously described by Grohs et al. (18). Pooled four-site surveillance samples were collected from the nares, axilla, umbilical stump, and inguinal region. Infants with suspected infection were also cultured by clinical providers as part of routine clinical care. The Columbia University Medical Center Institutional Review Board approved this study with a waiver of informed consent.

### Identification of S. aureus, antibiotic susceptibility testing, and decolonization strategies.

MSSA and MRSA isolates were identified and differentiated by culturing on both Columbia CNA (Becton, Dickinson) and chromogenic agar (Spectra MRSA; Remel), with confirmation via catalase and rapid latex agglutination (StaphAurex; Remel) and cefoxitin disk diffusion and the PBP2a culture colony test (Alere), as described in reference 17. Mupirocin resistance was assessed by gradient diffusion for isolates obtained by surveillance but not for isolates associated with clinical infection (Etest, bioMérieux). Mupirocin susceptibility was defined by a MIC of ≤4 μg/mL (38). Full antimicrobial susceptibility testing was performed on clinical but not surveillance isolates using the MicroScan WalkAway 96 Plus system with Pos MIC panel type 34 (Beckman Coulter).

As previously described, infants eligible for MSSA decolonization with chlorhexidine gluconate baths were >36 weeks gestational age or >4 weeks chronologic age (18). Eligible infants were bathed on days 1, 5, and 7 of their admission. Infants colonized with mupirocin-susceptible MSSA, regardless of age, had mupirocin applied to the nares thrice daily for 7 days. In total, 1,556 infants were admitted to the NICU during the study period; 211 were MSSA positive based on active surveillance. Clinical MSSA isolates were collected from an additional 60 infants during this time frame, bringing the total number of MSSA-positive infants to 271 (Fig. S1) (18).

### Genomic characterization and phylogenetic reconstruction of major MSSA clones.

For this study, 140 surveillance and clinical isolates (Fig. S1) were characterized by *spa*-typing using PCR amplification from single colonies with standard primers and Sanger sequencing, as previously described (18). We then sequenced all 125 surveillance and clinical isolates from the study period belonging to ST398 (*spa* t571 and t1451; *n* = 31 from 18 infants) and ST1898 (*spa* t279, *n* = 94 from 48 infants) (Fig. S1). These two *spa* types were isolated from the greatest number of infants throughout our NICU surveillance period. Of note, t571 and t1451 are associated with ST398, a prevalent MSSA clone in the community surrounding our NICU (19, 22). To provide additional context for the emergence of MSSA-t279 in the NICU, we also sequenced clinical isolates harboring t279 (*n* = 4 from 3 infants) and the closely related t228 (*n* = 11 from 9 infants), collected in the year prior to the initiation of the surveillance effort as a part of routine clinical care.

For WGS, library preparation was carried out using the Nextera DNA Flex library prep kit (Illumina), and sequencing was performed on an Illumina MiSeq instrument with a v3 600-cycle kit. SRST2 (39) was used for multilocus sequence typing and identification of known antibiotic resistance genes and plasmid replicons. WGS metrics are provided in Table S1.

10.1128/msphere.00409-22.1 TABLE S1 Whole-genome sequencing metrics for isolates included in this study. Download Table S1, XLSX file, 0.02 MB. Copyright © 2022 Annavajhala et al. 2022 Annavajhala et al. https://creativecommons.org/licenses/by/4.0/ This content is distributed under the terms of the Creative Commons Attribution 4.0 International license.

We generated a reference genome for MSSA ST1898, as no public reference genomes were available. An index NICU ST1898 isolate collected in 2016 (CUMC4945; NCBI Genome JAEHFK000000000) underwent long-read Oxford Nanopore sequencing on a MinION device after DNA extraction using the QIAamp DNA minikit (Qiagen) and library preparation using the rapid barcoding kit (SQK-RBK004, Oxford Nanopore) as previously described (40, 41). Hybrid assembly using Nanopore and Illumina reads was performed with Unicycler (42), and annotation was performed with Prokka (43). Integrated phage regions and genomic islands were predicted using PHASTER (44) and IslandViewer 4 (45).

Paired-end reads for each isolate were mapped using Snippy (46) against the appropriate reference genome (ST398 reference genome [20] CP003045.1 or ST1898 genome JAEHFK000000000). Mobile genetic elements, including integrated phage regions, were excluded, after which there was no evidence of recombinant sites as determined using Gubbins. The same approach was used to map publicly available short reads from 271 ST398 isolates collected from community households in northern Manhattan, near our tertiary care center (47). Serial isolates collected from the same individual were included in phylogenetic trees and in analysis of pairwise SNP distances within and across individuals. Core genome single nucleotide polymorphisms (SNPs) were concatenated to generate maximum-likelihood phylogenetic reconstructions for ST398 (5,338 concatenated SNP sites from a core genome size of 2,391,431 bp) and ST1898 with or without ST15 (81 concatenated SNP sites from a core genome size of 2,561,464 bp) isolates using RAxML (48) with 100 bootstrap replicates. Phylogenetic trees were visualized and annotated using iTOL (49).

### Data availability.

The genomic data sets generated and analyzed in the current study are available in the NCBI Sequence Read Archive (SRA) under BioProject PRJNA685142. The CUMC4945 genome used as a genomic reference for phylogenetic analyses of CC15 is available through the NCBI Genome repository (https://www.ncbi.nlm.nih.gov/genome/) under accession number JAEHFK000000000.

### Ethics, consent, and permissions.

The Columbia University Medical Center Institutional Review Board (IRB) approved this study with a waiver of informed consent.
