# Supplementary figures and images for: Genomic and Epidemiological Features of Two Dominant Methicillin-Susceptible Staphylococcus aureus Clones from a Neonatal Intensive Care Unit Surveillance Effort
Source: mSphere. 2022 Oct 11;7(6):e00409-22. doi: 10.1128/msphere.00409-22 (PMC9769867; doi:10.1128/msphere.00409-22)

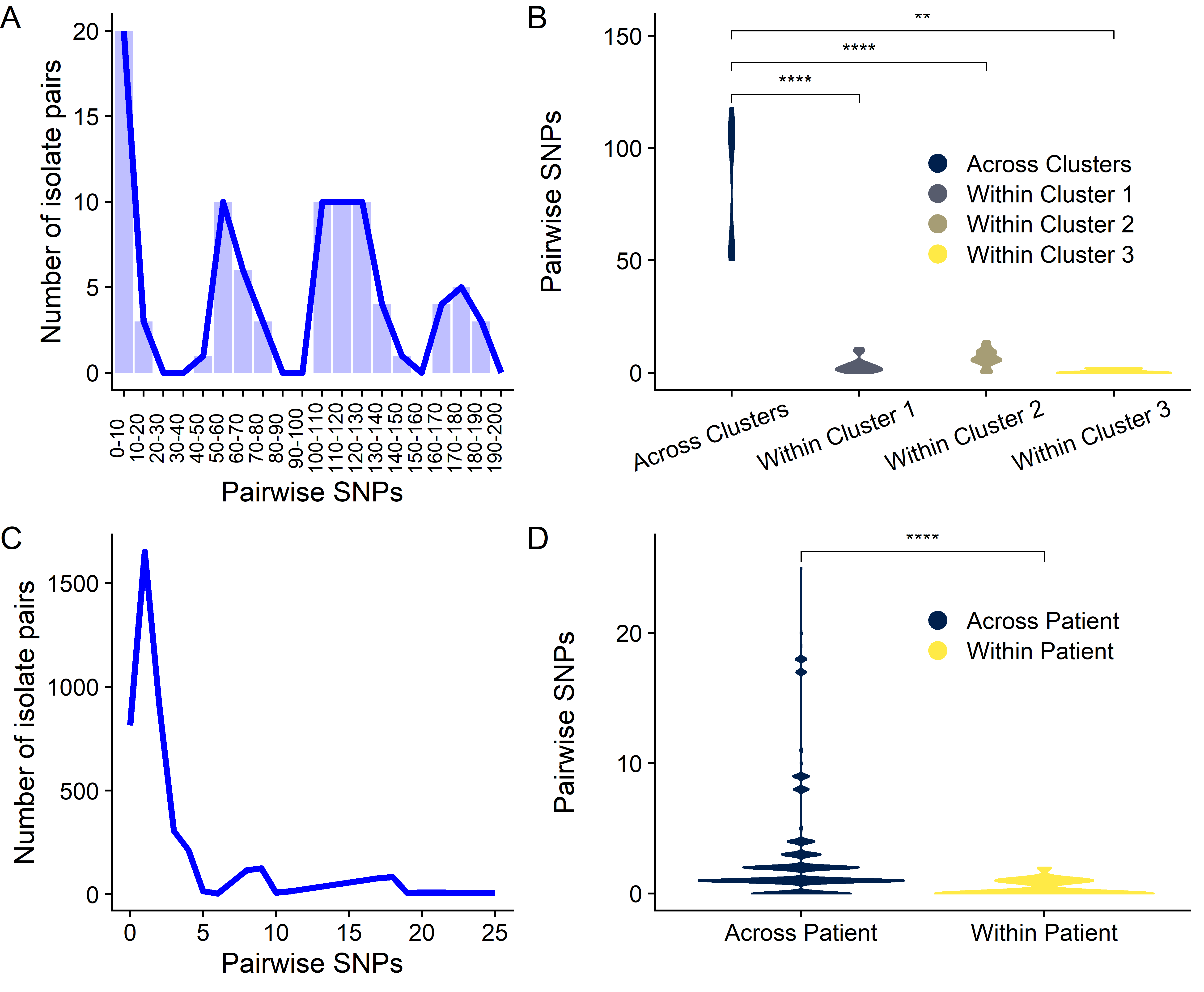

Supplement: FIG S2 [file msphere.00409-22-s0003.tif]

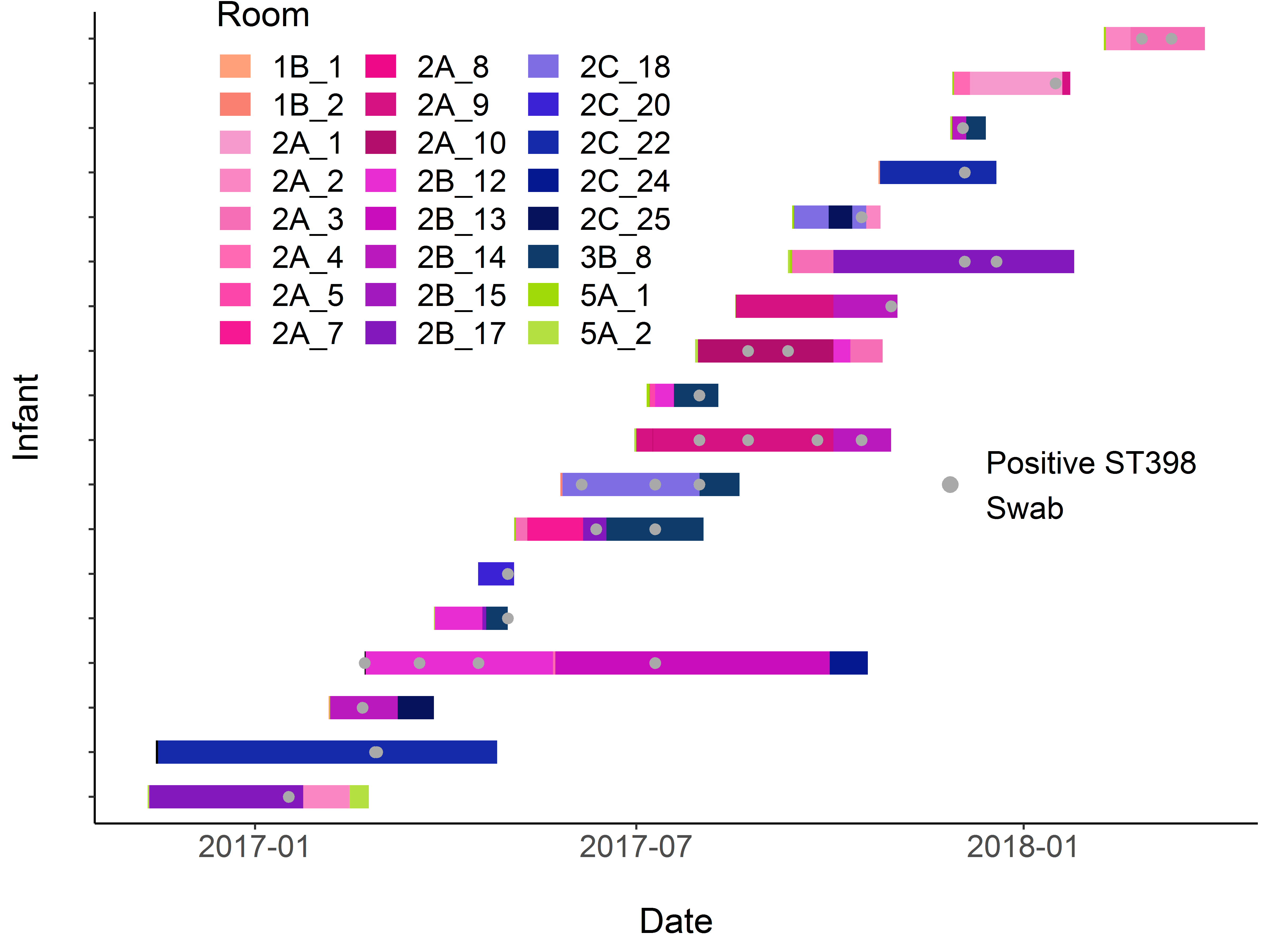

Supplement: FIG S3 [file msphere.00409-22-s0004.tif]

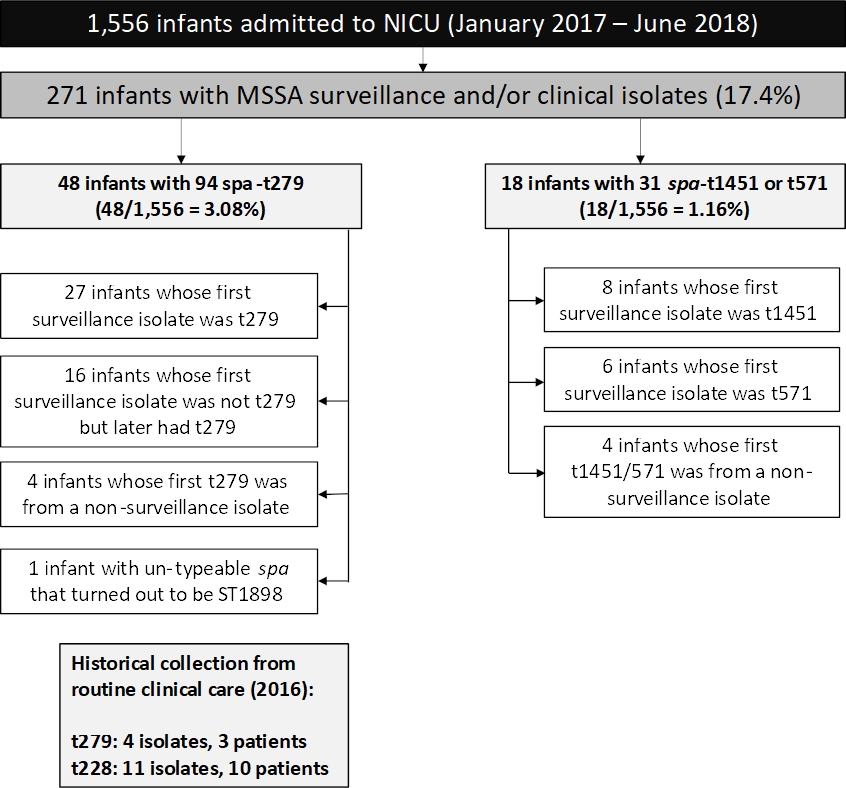

Supplement: FIG S1 [file msphere.00409-22-s0002.tif]

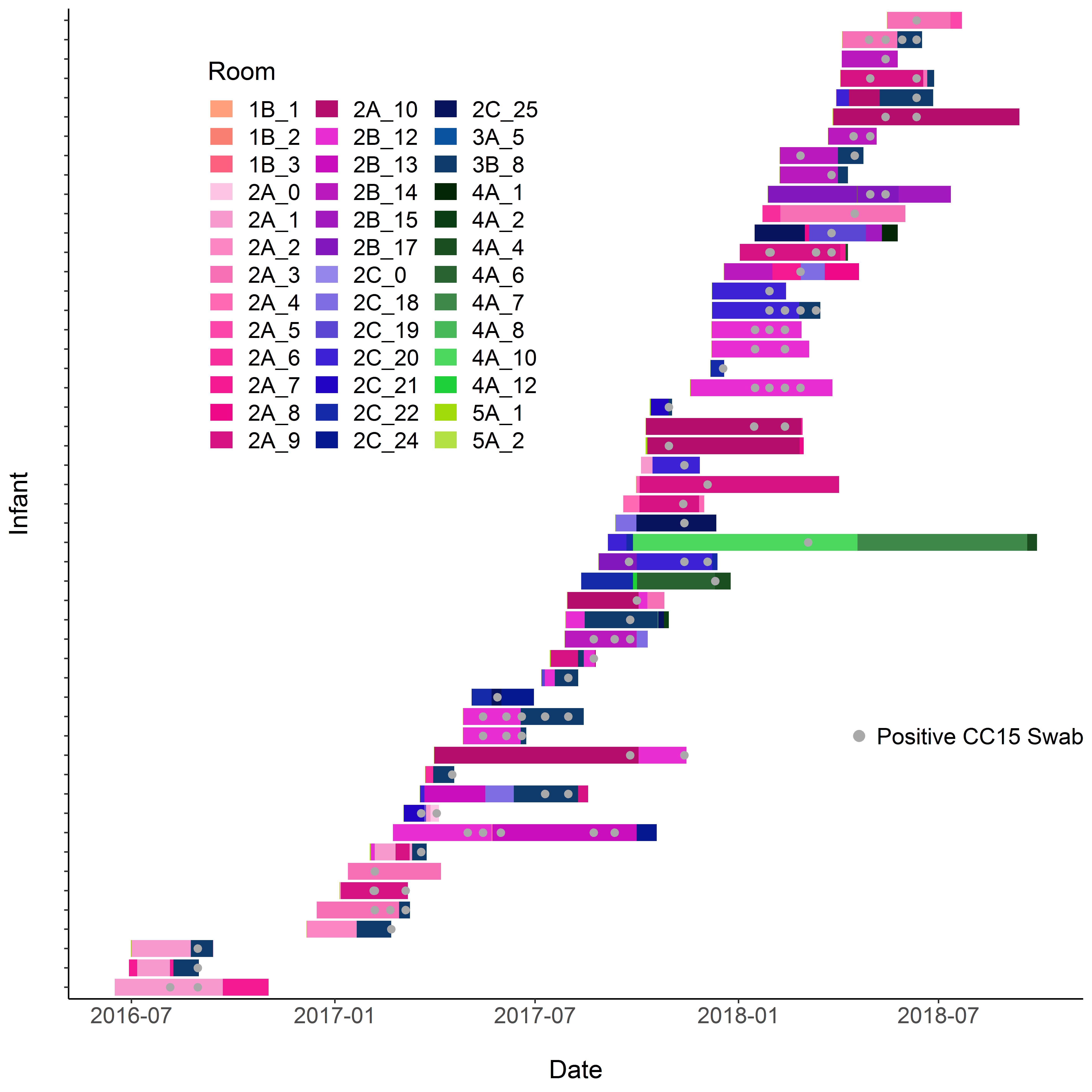

Supplement: FIG S4 [file msphere.00409-22-s0005.tif]

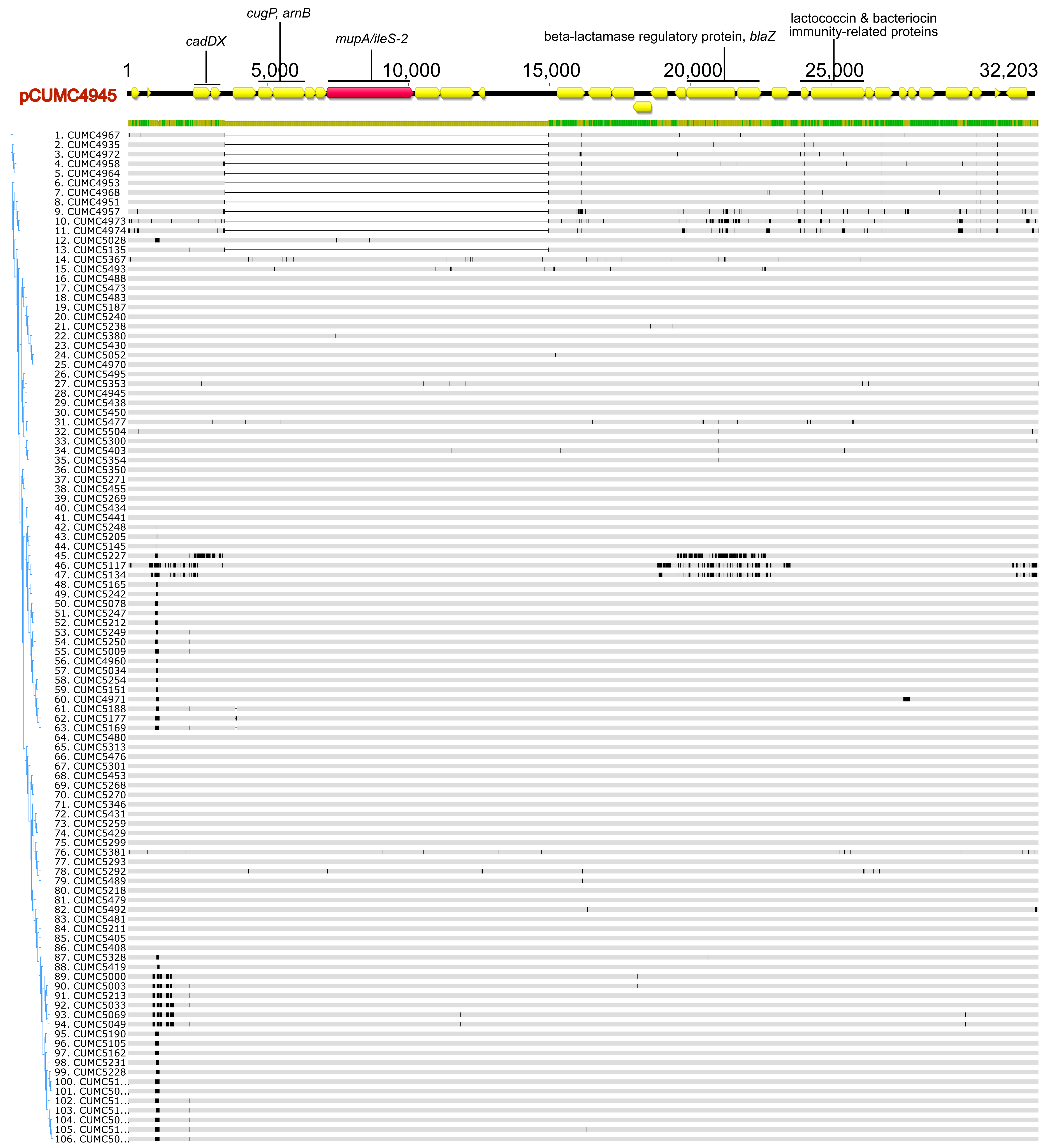

Supplement: FIG S5 [file msphere.00409-22-s0006.tif]
